# Olefin metathesis in air

**DOI:** 10.3762/bjoc.11.221

**Published:** 2015-10-30

**Authors:** Lorenzo Piola, Fady Nahra, Steven P Nolan

**Affiliations:** 1 EaStCHEM, School of Chemistry, University of St Andrews, St Andrews, KY16 9ST, UK; 2 Chemistry Department, College of Science, King Saud University, Riyadh 11451, Saudi Arabia

**Keywords:** air stability, catalysis, olefin metathesis, RCM, ROMP, ruthenium

## Abstract

Since the discovery and now widespread use of olefin metathesis, the evolution of metathesis catalysts towards air stability has become an area of significant interest. In this fascinating area of study, beginning with early systems making use of high oxidation state early transition metal centers that required strict exclusion of water and air, advances have been made to render catalysts more stable and yet more functional group tolerant. This review summarizes the major developments concerning catalytic systems directed towards water and air tolerance.

## Introduction

Transition metal-catalyzed alkene metathesis [1–10], which involves a fragment exchange between alkenes, is nowadays one of the most used strategies for the formation of carbon–carbon bonds. This area of study began with a “black box” approach for catalysts formation in polymerization of olefins. In recent years, metathesis-type reactions have emerged as universal strategies, employed in many fields of organic chemistry: from polymer chemistry [11–18] to natural product [19–21] and fine chemical syntheses [3,22–25]. Its importance led to the 2005 Nobel Prize in chemistry being awarded to Yves Chauvin, Richard Schrock and Robert Grubbs, who developed and studied this reaction [26]. Its wide adoption in organic reactions, where the use of inert and dry conditions are not always desirable, has led to efforts to develop new catalytic systems that enable this transformation in the presence of air and water [27]. However, this field of research has suffered a slow growth and only recently, an increasing number of research groups have started to seriously focus on testing metathesis catalysts in the presence of air and water. This is a way to gauge catalyst stability but also to potentially bring operational simplicity to this now widespead assembly strategy.

In this review, we summarize improvements associated with the stability of well-defined metathesis homogeneous systems towards the presence of air and water in the alkene metathesis and hopefully raise the awareness of the significant tolerance of standard metathesis catalysts to these conditions.

## Review

### Well-defined ruthenium catalysts

Although well-defined early transition metal-based catalysts formed the basis of early metathesis reactions and can be thought of as the forefathers of modern metathesis catalysts [27–30], these all showed poor tolerance towards air and water, because of their high oxophilicity [3,8–9,16,27]. To date, there are no examples of their use in the presence of air.

To overcome the sensitivity problems exhibited by early transition-based catalysts, late transition metals, which do not exhibit high oxophilicity, appeared as the most promising candidates for reactions performed in air.

**Scheme 1:**
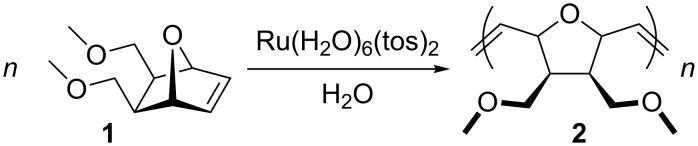
Polymerization of 7-oxanorbornene in water.

Indeed in 1988, Grubbs and Novak reported that not only ruthenium was an interesting candidate for olefin metathesis, but also that reactions were successfully conducted in water [31–32]. They discovered that Ru(H_2_O)_6_(tos)_2_ could polymerize 7-oxanorbonene **1** in water under air (Scheme 1).

In 1991, Marciniec and Pietraszuck reported the catalytic activity of RuCl_2_(PPh_3_)_3_ in the self-metathesis of silicon-contaning olefins. The reactions were performed with 1 mol % of Ru at 150 °C in air, under solvent-free conditions for several days, to afford 1,2-bis(silyl)ethenes in moderate to good yields [33]. Reactions without oxygen showed no conversion, highlighting the important role that the latter plays in the activation of the catalyst.

**Scheme 2:**
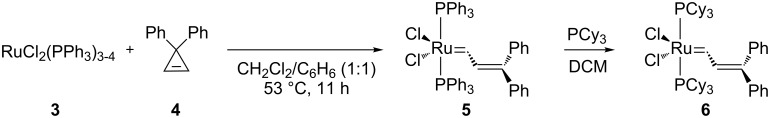
Synthesis of the first well-defined ruthenium carbene.

In 1992, Grubbs and co-workers synthesized the first well-defined ruthenium(II) complex (**5**, Scheme 2) bearing a carbene moiety, able to perform ring-opening metathesis polymerization (ROMP) reactions of low-strained olefins [34–35] and ring-closing metathesis (RCM) reactions of functionalized dienes [36]. In the solid state, this complex was reported to be indefinitely stable under inert atmosphere whereas it could survive for only several minutes in air. In solution, it was stable in several degassed organic solvents, even in the presence of water or HCl [35].

Exchanging PPh_3_ with PCy_3_ increased significantly the activity of the catalyst **6** (Scheme 2), which then was capable of polymerizing unstrained cyclic olefins and to perform reactions with acyclic olefins [37]. Subsequent variations showed that larger and more basic phosphine ligands led to improved activity, and that an order of activity could be established as PCy_3_ > P(iPr)_3_ >> PPh_3_. Reactions had to be performed in degassed and distilled solvents under N_2_ atmosphere to obtain maximum yields.

### Grubbs’ 1^st^ generation catalyst

**Scheme 3:**
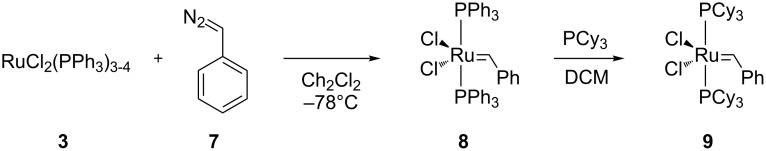
Synthesis of Grubbs' 1^st^ generation catalyst.

To overcome the aforementioned difficulties, Grubbs and co-workers synthesized, what has become known as the Grubbs’ 1^st^ generation catalyst (**9**, Scheme 3). The reaction of RuCl_2_(PPh_3_)_3-4_ (**3**) with phenyldiazomethane (**7**), followed by a phosphine exchange reaction, afforded complex **9** in high yields. Complex **9** has become the most used metathesis catalyst, because of its good activity, relatively good stability to air (storage of **9** has been recommended to be performed under anaerobic conditions and lower temperatures), compatibility with a large variety of functional groups [36,38] and because of its feasible large-scale production. So far, the use of this catalyst in air has not been reported.

### 2^nd^ generation catalyst

**Figure 1:**
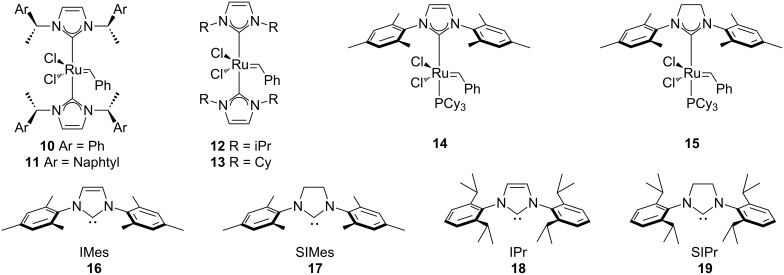
NHC-Ruthenium complexes and widely used NHC carbenes.

The synthesis of heteroleptic complexes, bearing one *N*-heterocyclic carbene (NHC) (**16–19**, Figure 1) and one phosphine as ligands, represented the second crucial turning point in this chemistry. Following Herrmann’s report on bis-NHC ruthenium complexes (**10–15**) and their low activity [39], independently and simultaneously the groups of Nolan (**14**) [40–41], Grubbs (**15**) [42–45] and Hermann [46–48] reported on the synthesis of this family of complexes. The combination of a labile phosphine group with a non-labile NHC ligand provided a significant improvement in terms of reactivity and stability. The bulky NHC provides steric protection to the metal center and its σ-donating ability stabilizes both the pre-catalyst and the catalytically operating intermediate [49]. The most active being complex **15**, bearing SIMes (1,3-bis(2,4,6-trimethylphenyl)-4,5-dihydroimidazol-2-ylidene, **17**) as ligand, is known nowadays as the Grubbs’ 2^nd^ generation catalyst. The increased stability of **17** is due to the unsaturated backbone of the NHC; the steric bulkiness on the metal center is improved and the σ-donating ability is increased compared to other NHCs.

These were the first ruthenium-based catalysts able to perform RCM reactions of tri- and tetrasubstituted olefins [42,46], cross-metathesis (CM) to afford trisubstituted olefins [44] and CM and RCM reactions of electron-withdrawing substituted olefins [45]. In comparison to the 1^st^ generation, they show a generally higher stability towards thermal degradation [41–43,49–50]. To date, only one example is reported where catalyst **15** is used in air (see following section).

### Hoveyda–Grubbs catalyst

**Scheme 4:**
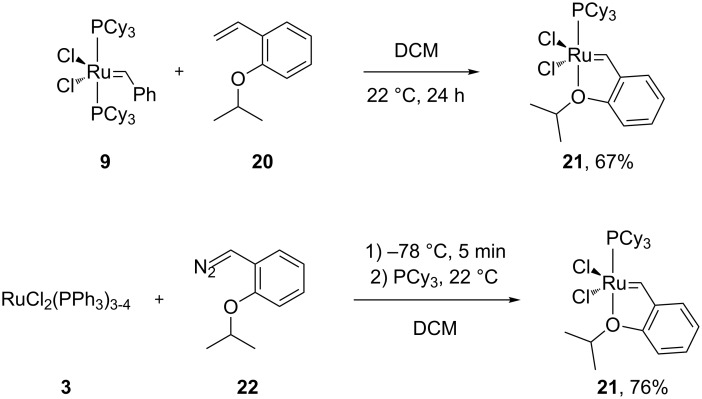
Access to **21** from the Grubbs’ 1^st^ generation catalyst and its one-pot synthesis.

The next notable evolution in terms of higher catalyst stability came from the Hoveyda group in 1999 [51]. While performing metathesis in the presence of isopropoxystyrene (**20**, Scheme 4), they noticed that the reaction proceeded very slowly. They postulated that the isopropoxystyrene formed a Ru-chelate complex in situ, which would be more stable than the precatalyst used in the reaction; therefore reducing the rate of the subsequent steps. Upon synthesis and evaluation of this new Ru-chelate complex (**21**, Scheme 4), they noted its astonishing stability. It could be recycled after reaction via column chromatography and it could be kept in undistilled CDCl_3_ for 2 weeks without any noticeable decomposition [51]. The isopropoxy group stabilized the complex by chelating the Ru moiety. Decomplexation of the latter allowed the approach of the olefinic substrate. Once the reaction reached completion and the starting materials depleted, the isopropoxy group coordinated back to the Ru center, allowing for the recycling of the catalyst. However, it should be mentioned that this increased stability diminished the activity of **21** when compared to **15** [52].

**Scheme 5:**
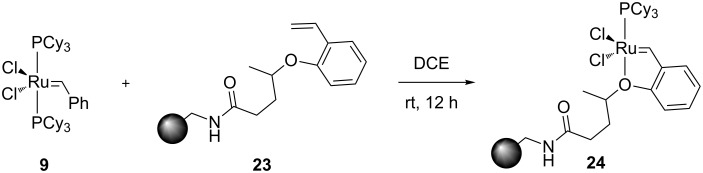
Synthesis of supported Hoveyda-type catalyst.

**Figure 2:**
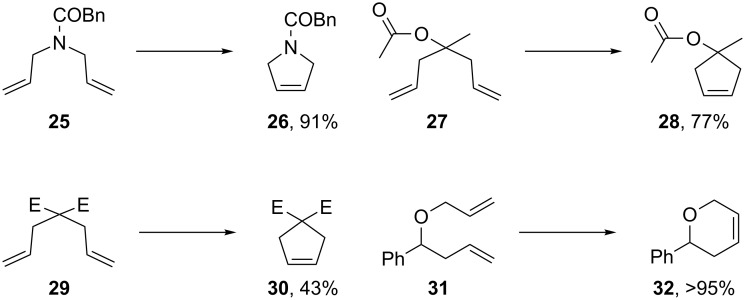
Scope of RCM reactions with supported Hoveyda-type catalyst. Reaction conditions: **24** (5 mol %), nondegassed DCM, rt, 3 h, in air. Conversions determined by ^1^H NMR. E = COOEt.

In 2000, Dowden [53] and co-workers reported the use of a polystyrene-supported ruthenium complex **24** (Scheme 5); a variation of the Hoveyda–Grubbs catalyst. It could be reused up to 5 times without loss of activity and without the use of a stabilizer. The catalysts were stored and used in air with non degassed DCM, providing average to good yields, with a catalyst loading of 5 mol % (Figure 2).

**Scheme 6:**
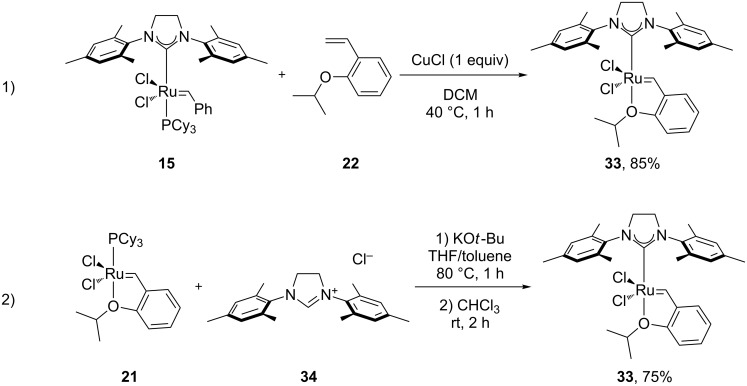
Synthesis of **33** by Hoveyda and Blechert.

As complex **21**, the efficiency of **24** is limited to terminal alkenes [54], and performs poorly in CM reactions. Soon after, in 2000, the Hoveyda–Grubbs 2^nd^ generation catalyst was reported (**33**), simultaneously, by Hoveyda (Scheme 6, entry 1) [54] and Blechert (Scheme 6, entry 2) [55] bearing a SIMes ligand instead of the phosphine.

Complex **33** was able to perform RCM of trisubstitued olefins and CM in high efficiency, and retained the properties of stability and recyclability.

**Figure 3:**
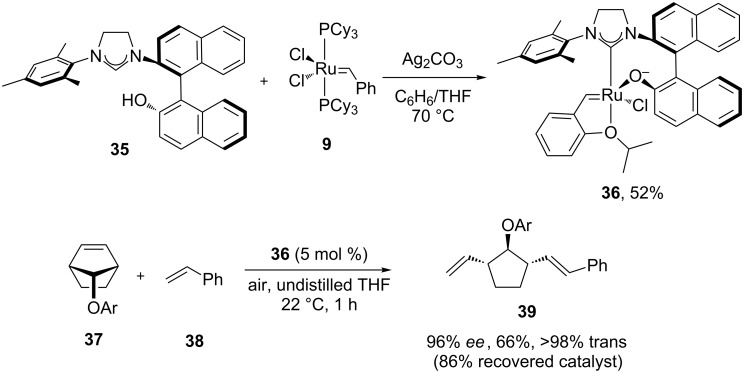
Synthesis of chiral Hoveyda–Grubbs type catalyst and its use in RO/CM.

In 2002, Hoveyda et al. reported the Hoveyda–Grubbs’ 2^nd^ generation type catalyst **36** (Figure 3) [56]: Complex **36**, bearing an unsymmetrical and chiral NHC, was active in the asymmetric ring-opening cross-metathesis (RO/CM) in air using undistilled solvents, and yielded products with high enantiomeric excess (ee). The results where comparable to previously reported results for molybdenum-catalyzed systems [57], although the latter was used under inert conditions.

**Scheme 7:**
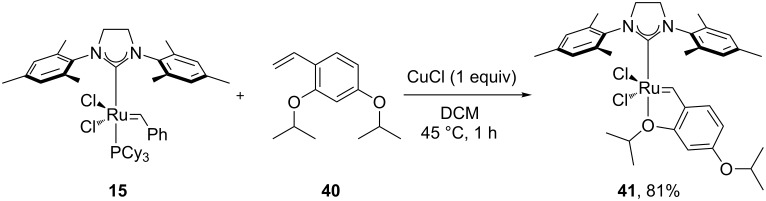
Synthesis of **41**.

In 2003, Blechert et al. reported the first systematic example of olefin metathesis in air [58]. Grubbs’ 2^nd^ generation catalyst **15** was compared to an *m*-isopropoxy-substituted Hoveyda–Grubbs’ 2^nd^ generation catalyst **41** (Scheme 7), using MeOH, water and DMF as solvents. Catalyst **41** bore two isopropoxy groups; the first one presented as a chelating group for the ruthenium center and the second one increased the solubility of the complex in alcohol solvents and DMF.

**Figure 4:**
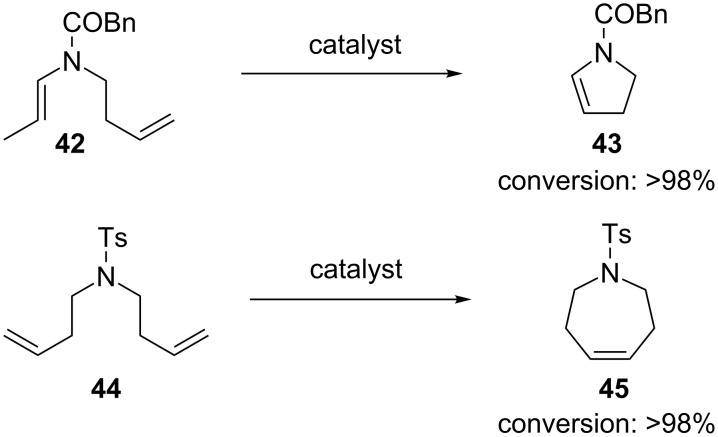
RCM reactions in air using **41** as catalyst. Reaction conditions: **41** (5 mol %), MeOH (0.05 M), 22 °C, 12 h, in air.

RCM reactions led to high conversions with all the solvents used, employing 5 mol % of **41** (Figure 4 and Table 1). It should be noted that catalyst **15** gave lower conversions when the water ratio was increased but it remained compatible with air.

**Table 1:** RCM in water and MeOH under air.^a^

Solvent	Substrate	Product	Conversion [%]^b^	
			**15**	**41**

MeOH MeOH/H_2_O (3:1) MeOH/H_2_O (1:1)^c^ MeOH/H_2_O (1:3)^c^	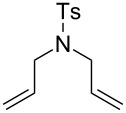 **46**	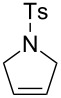 **47**	94 29 54 77	96 87 90 94

^a^Reaction conditions: Catalyst **15** or **41** (5 mol %), undistilled solvent (0.05 M), 22 °C, 12 h, in air. ^b^Determined by ^1^H NMR spectroscopy. ^c^Substrate not miscible with solvent [58].

**Figure 5:**
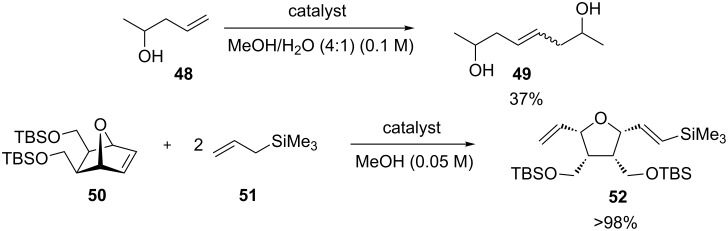
CM-type reactions in air using **41** as catalyst. Reaction conditions: **41** (5 mol %), 22 °C, 12 h, in air.

The CM reaction, which is known to be a most difficult reaction, gave only low yield, while the ROM/CM reaction gave a much higher yield (Figure 5). It should be noted that long reaction times were needed as well as high catalyst loadings (5 mol %) in these transformations.

In 2004, the Grela group presented some variations of the Hoveyda–Grubbs catalyst **21** [52,59–60]. They reported some modifications to the isopropoxystyrene group; a nitro group *para* to the isopropoxy moiety of the carbene provided a much faster initiating catalyst (**87**, Figure 12) than **21**, due to the weakening of the O–Ru bond [59–61]. Its use in air was reported by Olszewski, Skowerski and co-workers in a comparison with other catalysts (see section on indenylidene complexes, below) [62].

**Figure 6:**
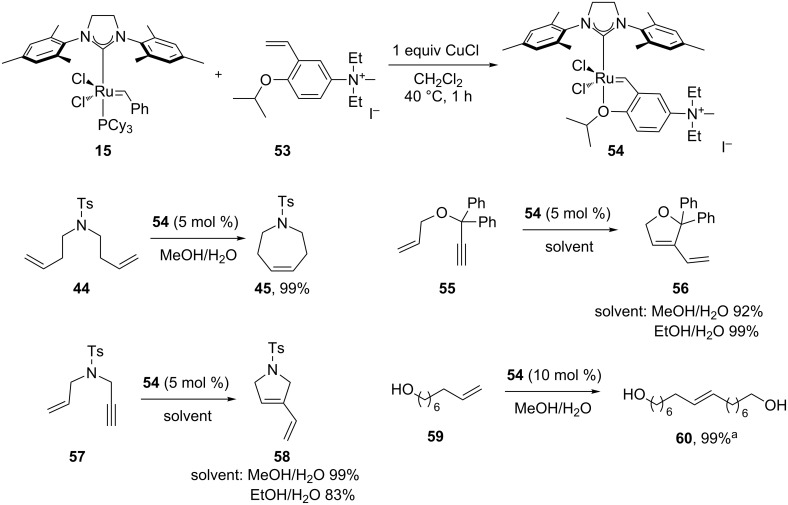
Grela's complex (**54**) and reaction scope in air. Reaction conditions: catalyst, substrate (0.25 mmol), nondegassed solvent (5:2; 0.02 M), 25 °C, in air, 0.5 h. GC conversion. ^a^Reaction time 24 h.

Soon after, in 2006, the same group presented a variation of the Hoveyda–Grubbs 2^nd^ generation catalyst, bearing a quaternary ammonium group (**54**, Figure 6) [63]. Complex **54** was used in nondegassed mixtures of MeOH/EtOH and water giving complete conversions in most cases, with short reaction times; although, requiring a high catalyst loading (5 mol %). The quaternary group increased the solubility in solvent mixtures and also increased the activity of the complex due to the electron-withdrawing effects of substituents.

In early 2009, Grubbs and co-workers reported the use of Hoveyda–Grubbs 2^nd^ generation catalyst **33** (0.1 mol %) in air and in different solvents for the RCM of diethyl diallylmalonate (**29**) [64]. Conversions were found to be as low as 10% in DCM and <20% in toluene.

**Figure 7:**
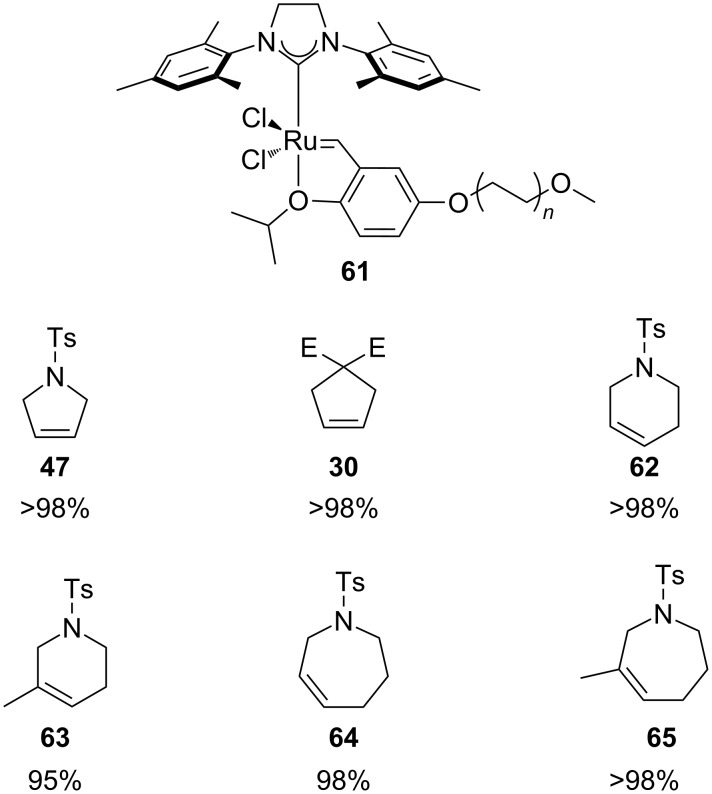
Abell's complex (**61**) and its RCM reaction scope in air. Reaction condition: 10 mol % of **61**, refluxing DCM in air, 0.5 h. Conversion determined by ^1^H NMR.

In 2009, Abell and Zaman reported the use of a Hoveyda–Grubbs 2^nd^ generation ruthenium-based catalyst immobilized on PEG (**61**, Figure 7) [65]. This catalyst was soluble in dichloromethane but could be retrieved and recycled by simple exctraction with water or precipitation with ether. With a catalyst loading of 10 mol % in refluxing nondegassed dichloromethane, very high conversions were achieved in less than 1 hour for di- and trisubstituted olefins.

**Figure 8:**
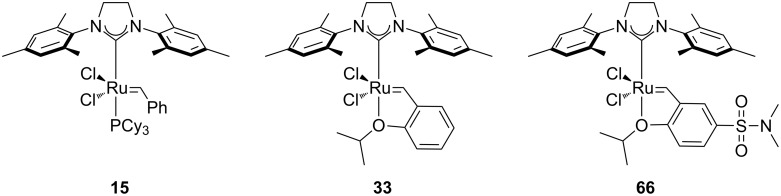
Catalysts used by Meier in air.

Towards the end of 2009, the Meier group reported the use of Grubbs (**15**), Hoveyda–Grubbs 2^nd^ generation catalyst (**33**) and a variation of the latter (**66**, Figure 8) in the RCM of diethyl diallylmalonate (**29**) [66]. Reactions were performed with very low catalyst loading (from 2.5 to 0.04 mol %), at 30 °C, under air in nondegassed DCM, nondegassed methyl decanoate and under solvent-free conditions in nondegassed substrates. Full conversions were achieved in the majority of cases, in both CM and RCM reactions, with all catalysts. In these reactions, catalyst **66** gave the highest performance. It should be noted that the results obtained by Meier with **33** were in contrast with the previous report by Grubbs [64].

**Figure 9:**
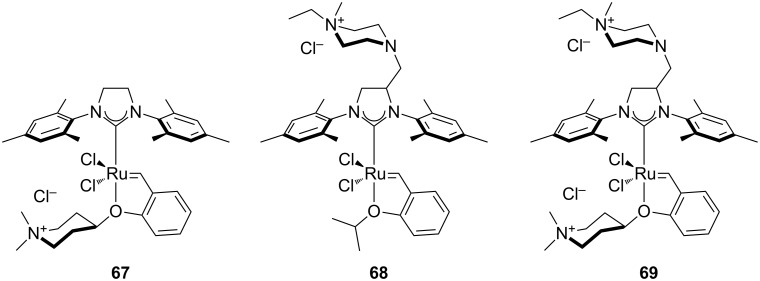
Ammonium chloride-tagged complexes.

In 2012, Grela and co-workers described the synthesis and use of 3 ammonium chloride-tagged variations of Hoveyda–Grubbs’ catalyst (**67–69,** Figure 9) [67]. The catalysts were active in the isomerization of double bonds, self-metathesis, RCM and ene–yne metathesis reactions. They afforded average to high yields under air (Table 2). Reactions were performed in water at rt. Catalyst **69** was the most soluble in water; however, it did not afford the highest catalytic activity. In order to test the recyclability of the complex, diethyl diallymalonate (**29**) was subjected to RCM reaction in refluxing DCM with 1 mol % of catalyst **69**. After reaction completion (97% isolated yield) and a single extraction with D_2_O, (*Z*)-but-2-ene-1,4-[^2^H]-diol was added to the water phase and isomerization to the *trans* isomer **71** was completeted after 1 h, with no decrease in activity (94% isolated yield) observed.

**Table 2:** Metathesis reaction in water under air.

Substrate	Product	Catalyst (mol %)	Time (h)	Yield (%)^a^

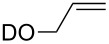 **70**	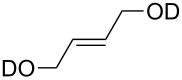 **71**	**67** (5) **68** (5) **69** (5)	24 24 24	74^b^ 77^b^ 38^c^

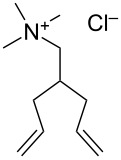 **72**	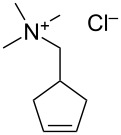 **73**	**67** (2.5) **68** (2.5) **69** (2.5)	3.5 2.5 2.5	49 96 88

^a^Yields are calculated by NMR spectroscopy. ^b^*E*/*Z* = 16.7:1. ^c^*E*/*Z* = 12.5:1 [67].

In early 2013, Jensen and co-workers reported a variation of the Hoveyda–Grubbs’ 2^nd^ generation catalyst bearing a sulfur-based anion (2,4,6-triphenylbenzenthiolate), replacing one of the chlorides [68]. Despite being a stable and a high *Z*-selective catalyst, it displayed no activity in air, using 0.01 mol % catalyst loading.

**Figure 10:**
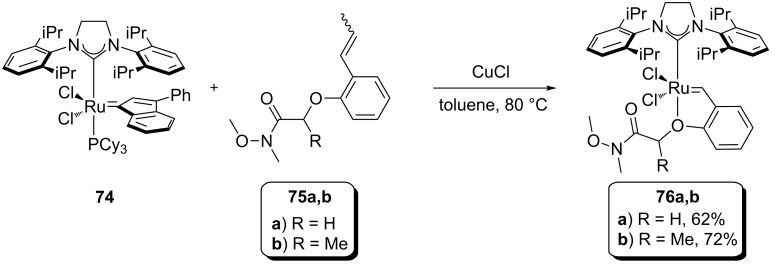
Scorpio-type complexes.

Later in the same year, Olszewski, Skowerski et al. reported the synthesis and use of new Scorpio-type complexes (Figure 10) [69]. These complexes presented high affinity for silica, which allowed the easy separation and recycling of the catalysts from the reaction mixture. Due to air stability, their activity in nondegassed DCM, toluene and ACS grade ethyl acetate was reported (Table 3). Complex **76b** performed slightly better in all cases, regardless of the air atmosphere and of the solvent used. With low catalyst loadings, ranging from 1 to 0.1 mol %, high to quantitative yields were achieved in all cases.

**Table 3:** Metathesis reactions catalysed by Scorpio-type complexes in air.^a^

Substrate^b^	Product^b^	Solvent (M)	Catalyst (mol %)	Time (min)	Yield (%)^c^

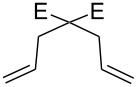 **29**	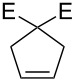 **30**	DCM (0.05) DCM (0.05) EtOAc (0.1 M)^d^	**76a** (1) **76b** (1) **76b** (0.2)	60 30 60	94 98 >99^e^

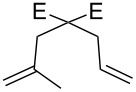 **77**	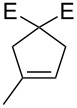 **78**	DCM (0.05) DCM (0.05)	**76a** (1) **76b** (1)	150 60	97 96

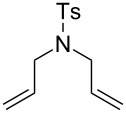 **46**	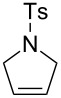 **47**	DCM (0.05) toluene (0.1)^f^	**76b** (1) **76b** (0.1)	20 60	>98 94

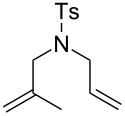 **79**	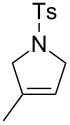 **80**	DCM (0.05) toluene (0.1)^f^	**76b** (1) **76b** (0.1)	40 60	>98 96

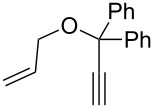 **55**	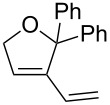 **56**	DCM (0.05) DCM (0.05) toluene (0.1)	**76a** (1) **76b** (1) **76b** (0.5)	45 30 300	99 <98 92

^a^Reaction conditions: catalyst, nondegassed DCM, reflux, *t*. ^b^E = COOEt. ^c^Isolated yields after column chromatography. ^d^Ethyl acetate is ACS grade solvent, temperature is 40 °C. ^e^Conversion determined by GC. ^f^Added dropwise with a syringe pump [69].

### Grubbs 3^rd^ generation catalyst

**Scheme 8:**
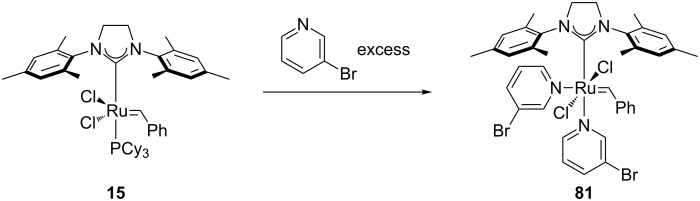
Synthesis of Grubbs' 3^rd^ generation catalyst.

In 2002, Grubbs’ and co-workers reported a variation of the 2^nd^ generation catalyst, featuring the substitution of PCy_3_ with two molecules of 3-bromopyridine (Scheme 8) [70]: Catalyst **81**, now known as Grubbs’ 3^rd^ generation catalyst, showed the highest rate of initiation reported to date for alkene metathesis reactions.

Complex **81** is used mostly for ROMP and CM reactions with electron-deficient olefins. The complex can be prepared in air but only one example of its use in air has been reported. In 2010, Tew and co-workers reported the use of **81** in the living ROMP of a hydrophilic norbornene monomer in air, leading to the formation of hydrogels [71]. Despite the living character of this reaction, the propagating catalyst was found to be inactive after 1 hour.

### Indenylidene complexes

**Figure 11:**
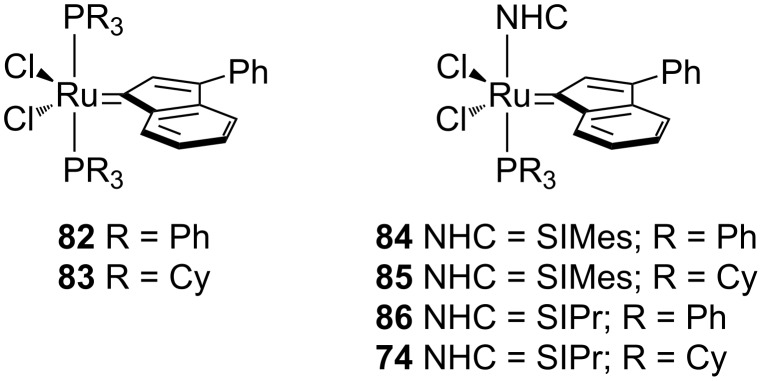
Indenylidene complexes.

The indenylidene-bearing family of complexes has exhibited a rapid growth in use in recent years and is quickly becoming a mainstream catalyst in metathesis-type reactions (Figure 11). These complexes have received significant attention due to their high activity in olefin metathesis [72–78], their thermal stability and their ease of synthesis [77,79–80].

Complex **82** is air-stable in the solid state; however, it does not show activity in metathesis-type reactions. On the other hand, its PCy_3_ counterpart **83** is as active as the Grubbs’ 1^st^ generation catalyst [73,80–81]. The NHC-bearing complexes (**74, 84–86**) showed increased activity and maintained the same thermal stability. Again, these complexes showed similar activity to the Grubbs 2^nd^ generation catalysts [77–78], and are stable when stored under air. Nolan reported the synthesis of Grubbs’ 2^nd^ generation catalyst (**15**) from indenylidene complexes **84**, by simple reaction with styrene, avoiding the use of hazardous diazo compound **7** [82].

**Figure 12:**
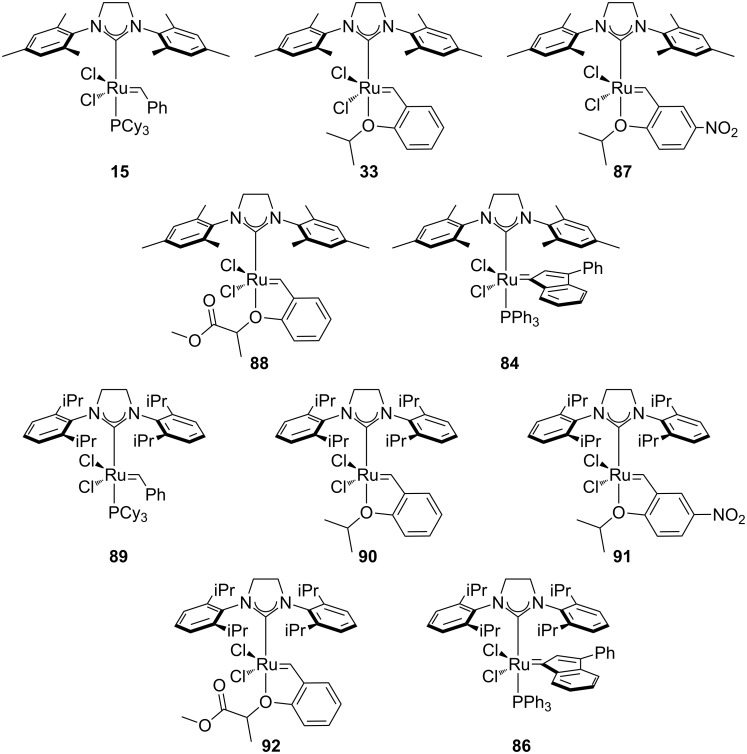
Commercially available complexes evaluated under air.

Towards the end of 2013, a report by Olszewski, Skowerski and co-workers showed how a variety of commercially available catalysts (Figure 12) could be employed in air with nondegassed ACS grade green solvents. Their results were in line with the ones obtained with DCM and toluene [62]. From Table 4, it can be seen how ethyl acetate at 70 °C represented an optimal solvent choice for most of the complexes.

**Table 4:** RCM with commercially available catalysts in technical grade solvents.^a^

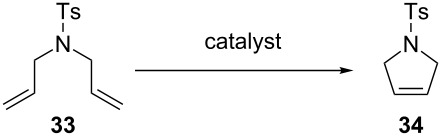						

		GC yield (%)				
Catalyst	*T* (°C)	AcOEt	DMC	CPME	2-MeTHF	DCM/toluene^b^

**15**	40	97	98	80	35	92
	70	98	98	97	95	67
**87**	40	94	85	79	49	96
	70	98	98	97	65	65
**88**	40	66	79	20	37	98
	70	99	98	60	65	61
**84**	40	96	98	69	38	93
	70	98	98	95	92	59
**89**	40	88	98	85	84	91
	70	99	98	92	97	98
**91**	40	96	99	97	97	88
	70	99	99	99	99	99
**92**	40	98	99	97	97	91
	70	99	99	99	98	99
**86**	40	92	98	89	93	95
	70	94	98	84	98	96

^a^Reaction conditions: Cat. 0.25 mol %, nondegassed, undistilled ACS grade solvents in air (0.1 M), 1 h. DMC: dimethyl carbonate; CPME: cyclopentyl methyl ether; 2-MeTHF: 2-methyltetrahydrofuran.^b^DCM was used at 40 °C while toluene at 70 °C [62].

Every catalyst afforded very high yields, in air, with activities comparable to the use of distilled and anhydrous solvents. Also reported was the cyclization of *N*-allyl-*N*-(methallyl)tosylamide (**79**) in nondegassed and undistilled ethyl acetate (ACS grade), catalyzed by **87** (0.25 mol %), at 70 °C in 1 h with a conversion of 98%.

**Figure 13:**
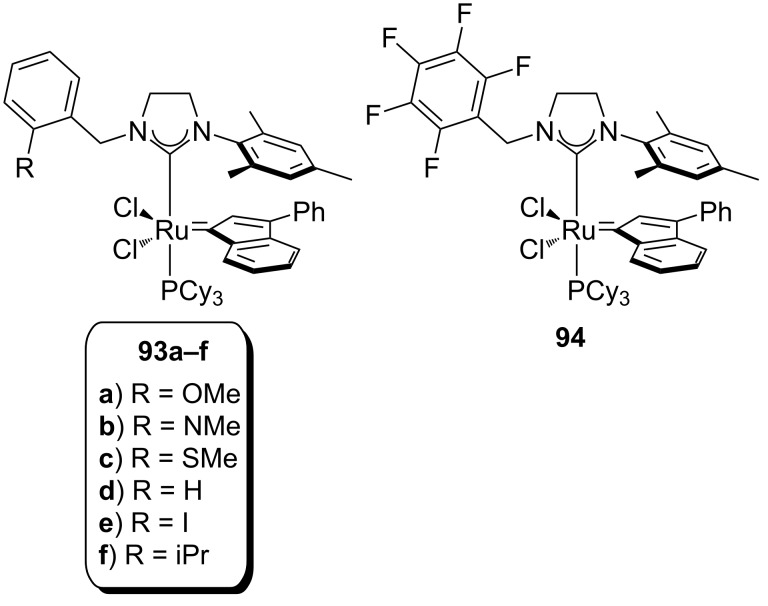
Grela's *N,N*-unsymmetrically substituted complexes.

In 2014, Grela and co-workers reported the synthesis *N,N*-unsymmetrically substituted SIMes-bearing indenylidene complexes (**93a–f** and **94**, Figure 13) [83]. They also tested their reactivity under air and in technical grade nondegassed solvents, and compared them to the activity of the commercially available catalyst **85**.

After initial screening and evaluation of their activity with the model substrate, diethyl diallylmalonate (**29**) (Table 5), **93a**, **93b**, **93d** and **93e** were found more active than **85**. When diethyl allyl(methallyl)malonate (**77**) and *N*,*N*-bis(methallyl)tosylamide (**95**) were used, catalysts **93a** and **93b** performed better than others. A full scope, involving an ene–yne reaction, was carried out with these two complexes in DCM and toluene in comparison with **85**; catalyst loadings were between 1 and 2 mol % and reaction times, with the synthesised complexes, were shorter than with **85**.

**Table 5:** RCM and ene–yne reactions catalysed by **93a–f** and **94** in air.^a^

Substrate	Product^b^	Catalyst (mol %)	*T* (°C)^c^	*t* (h)	Yield (%)^d^

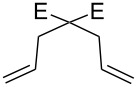 **29**	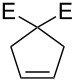 **30**	**85** (1) **93a** (1) **93b** (1) **93c** (1) **93d** (1) **93e** (1) **93f** (1) **94** (1)	30 30 30 30 30 30 30 30	0.4 1.7 1.7 1.7 1.8 1.7 1.9 0.9	42 96 93 7 92 97 17 90

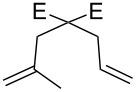 **77**	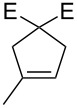 **78**	**85** (1) **93a** (1) **93b** (1) **93d** (1) **93e** (1)	30 30 30 30 30	1.7 1.7 1.5 1.7 1.5	23 87 72 72 86

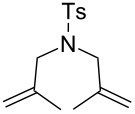 **95**	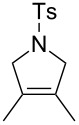 **96**	**85** (5) **93a** (5) **93b** (5) **93d** (5) **93e** (5)	30 30 30 30 30	0.4 0.4 0.4 0.4 0.4	40 41 38 36 35

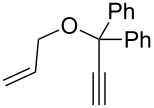 **55**	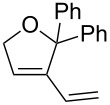 **56**	**85** (2) **93a** (2) **93b** (2)	30 30 30	6 5 8	94^e^ 96^e^ 96^e^

^a^Reaction conditions: Catalyst (mol %), nondegassed DCM (commercial-grade HPLC) (0.1 M) in air. ^b^E = COOEt. ^c^Reactions at 50 °C were performed in nondegassed toluene (commercial-grade HPLC) in air. ^d^Yields determined by ^1^H NMR. ^e^Isolated yields after flash chromatography [83].

### Phosphite-based catalysts

**Scheme 9:**
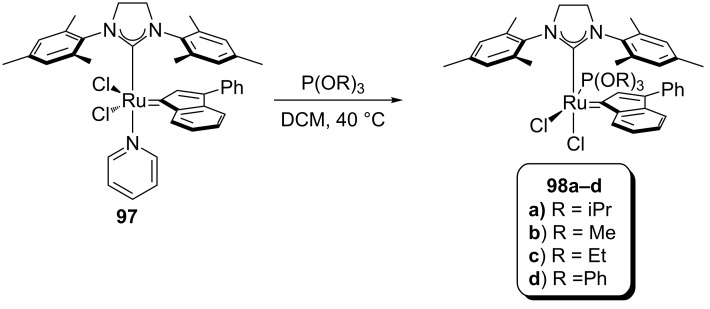
Synthesis of phosphite-based catalysts.

In 2010, the Cazin group reported a study on the synthesis and activity of a new family of complexes (**98a–d**, Scheme 9) [84]; phophite-based complexes were thus synthesized to evaluate possible positive effects of these ligands in alkene metathesis reactions.

Their stability at high temperatures allowed their use in the RCM of bis(methallyl)tosylamide (**95**) and diethyl bis(methallyl)malonate leading to the highest yields reported to date [85].

**Figure 14:**
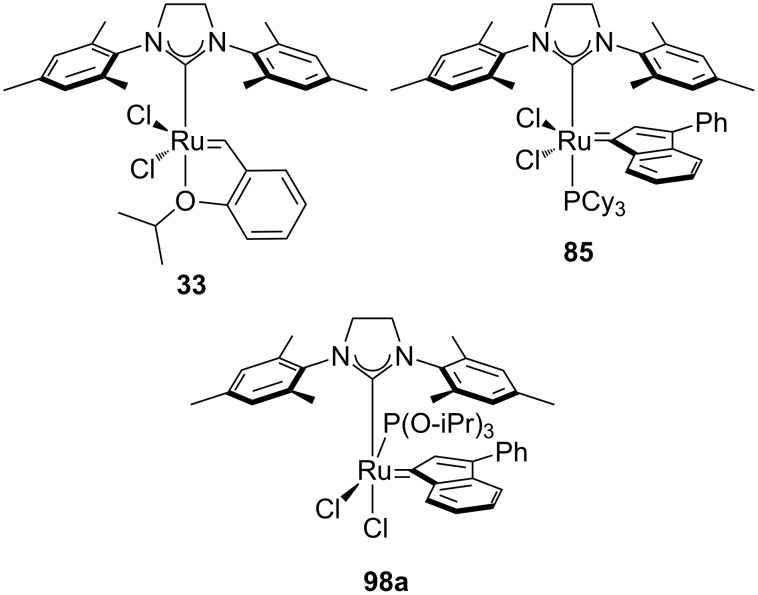
Catalysts used by the Cazin group.

In 2015, the same group reported a study on the use of **98a** and other commercially available metathesis catalysts (**15**, **33**, **85**, Figure 14) [86], under various conditions. Reactions were performed under atmospheres of N_2_, O_2_, CO_2_, air, dry air and in the presence of water to evaluate the effect of each on the performance of these catalysts.

A preliminary test on the RCM of bis(methallyl)tosylamide (**95**), using 0.1 mol % of **33**, **85** and **98a** under air and in refluxing toluene, showed a 60% conversion after 20 min for **98a**. Under these conditions, the other catalysts were completely inactive after 20 min and lead to conversions lower than 40%, when used for prolonged reaction times. After evaluation of the detrimental effects of each of the components of air on catalyst activity, a general trend could be observed: H_2_O > CO_2_ ≥ O_2_. In all cases, water had the most deleterious effect, whereas reactions could be performed in dry air and in N_2_ atmosphere without any noticeable differences as compared to their use under inert atmosphere.

**Figure 15:**
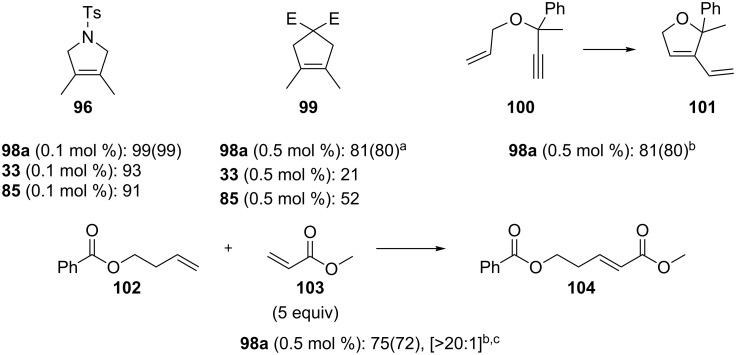
RCM scope in air with catalysts **33, 85** and **98a**. Reaction conditions: Catalyst, substrate (0.25 mmol), reagent-grade toluene (0.5 mL), 110 °C, in air, 3 h. E = COOEt. GC conversion and isolated yield in parentheses. ^a^Isolated as a mixture, NMR yield. ^b^Toluene (0.5 mL). ^c^*E*/*Z* ratio determined by ^1^H NMR.

Catalyst **98a**, with concentrations ranging from 0.05 to 0.5 mol %, exhibited the most remarkable activity in air with high to quantitative yields in the RCM, CM and ene–yne reactions. Furthermore, complexes **33** and **85** were able to perform the RCM reactions under the same conditions, with yields ranging from moderate to excellent (Figure 15).

### Schiff bases

Schiff bases in metathesis are usually O,N-bidentate ligands and represent an interesting alternative family of ligands as [18,87–94]: 1) they can be produced in one high yielding step by condensation of an aldehyde and an amine, thus allowing the fine and facile tuning of ligand and catalyst steric and electronic properties; and 2) the two different donor atoms, O (hard) and N (soft), offer different features and therefore can stabilize, respectively, high and low oxidation states.

**Figure 16:**
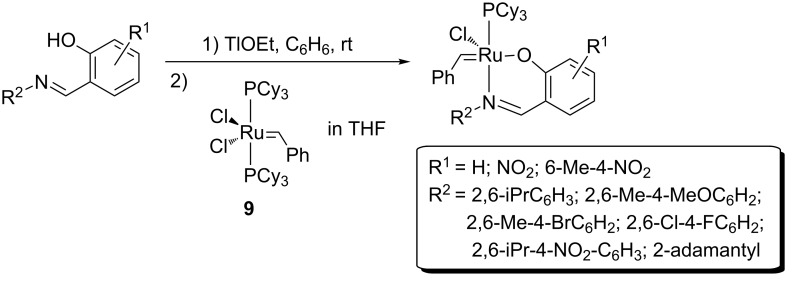
Synthesis of Schiff base-ruthenium complexes.

Ruthenium carbene complexes bearing Schiff bases were synthesized originally by the Grubbs’ group and applied in RCM reactions [95], showing lower activity then the Grubbs 1^st^ generation catalyst but exhibited very high termal stability (Figure 16).

**Scheme 10:**
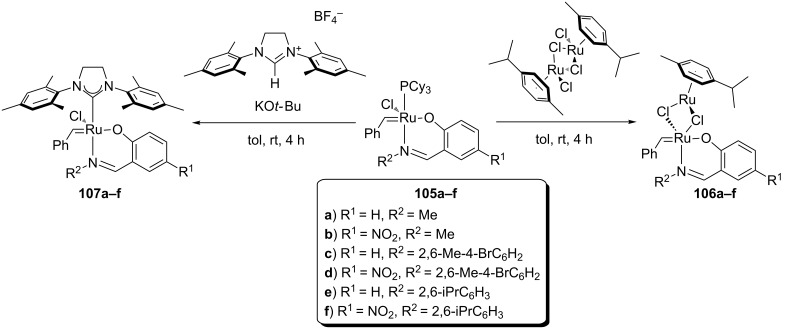
Schiff base–ruthenium complexes synthesized by Verpoort.

In 2002 and 2003, the Verpoort group synthesized and applied a variety of Schiff base adapted complexes in RCM [87] and ROMP [87,93–94,96–97] reactions (Scheme 10). This class of complexes showed high activity and very high stability to air and water, compared to Grubbs 1^st^ and 2^nd^ generation catalysts [7]. RCM reactions were performed in air with 5 mol % of the catalyst, showing high yields for terminal dienes (Table 6, entry 1). In the absence of SIMes, increasing the olefin substitution led to low yields in all catalytic systems. An electron-withdrawing substituent on the phenyl ring and a bulky group on the imine generally lead to higher activity for both mono- and bimetallic systems. SIMes-bearing complexes are more active than monometallic systems in all cases, and more active than bimetallic systems only when the iminic substituent is less bulky (Table 6, entries 2 and 3).

**Table 6:** Yield (%) of RCM reactions using catalysts **105a–f**, **106a–f** and **107a–f** in air.^a^

Entry	Product^b^	Yield (%)					
		**105a**/**106a**/**107a**	**105b**/**106b**/**107b**	**105c**/**106c**/**107c**	**105d**/**106d**/**107d**	**105e**/**106e**/**107e**	**105f**/**106f**/**107f**

1	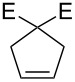 **30**	100/100/100	100/100/100	100/100/100	100/100/100	100/100/100	100/100/100
2	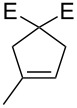 **77**	<5/13/72	<5/<5/73	<5/58/47	9/44/42	18/83/31	21/72/23
3	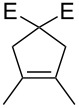 **99**	<5/6/41	<5/<5/33	<5/41/19	6/29/11	11/62/<5	17/49/<5

^a^Reaction conditions: catalyst (5 mol %), distilled C_6_D_5_Cl (0.05 M), 55 °C, in air for catalysts **105a–f** and 70 °C for catalysts **106a–f**, 4 h [96]. For catalysts **107a–f** undistilled C_6_D_6_ was used as solvent and temperature was 55 °C, 4 h, in air [97]. ^b^E = COOEt.

**Scheme 11:**
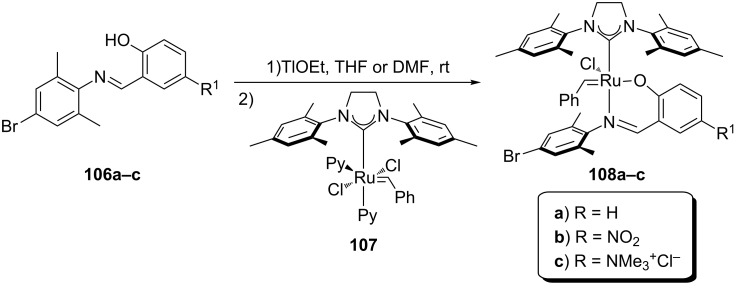
Synthesis of mixed Schiff base–NHC complexes.

In 2007, Raines et al. reported that **108b** (Scheme 11) remained intact after 8 days in C_6_D_6_ under air [7]. This prompted them to explore the activity of mixed Schiff–NHC complexes in RCM and ene–yne reactions using protic solvents in air.

As can be seen from Table 7, catalyst **108c**, bearing a water-soluble tag, is active in D_2_O and in water/methanol mixtures under air and the presence of the tag does not influence the reactivity. Although high conversions were obtained, high catalyst loadings (5–10 mol %) of all catalysts were required.

**Table 7:** RCM of representative dienes catalysed by **108a–c** under air.^a^

Substrate^b^	Product^b^	Solvent (substrate conc. [M])	Complex (mol %)	Time [h]	Conversion [%]^c^

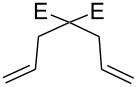 **29**	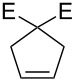 **30**	C_6_D_6_ (0.1) C_7_D_8_ (0.05) CD_3_OD (0.025) C_6_D_6_ (0.05)	**108a** (5) **108b** (5) **108b** (5) **108c** (5)	72 70 23 40	90 79 94 >95

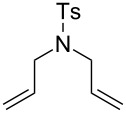 **46**	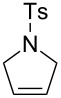 **47**	C_6_D_6_ (0.05) C_7_D_8_ (0.05) CD_3_OD (0.025) CD_3_OD (0.05) 2:1 CD_3_OD/D_2_O (0.025)	**108a** (5) **108b** (5) **108b** (5) **108c** (5) **108c** (5)	26 70 9 6 6	68 92 >95 >95 93

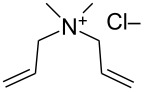 **109**	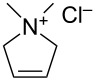 **110**	CD_3_OD (0.05) 2:1 CD_3_OD/D_2_O (0.025)	**108c** (5) **108c** (10)	12 6	79 40

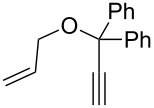 **55**	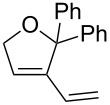 **56**	C_7_D_8_ (0.05) C_7_D_8_ (0.05) CD_3_OD (0.025) C_6_D_6_ (0.05) CD_3_OD (0.05)	**108a** (5) **108b** (5) **108b** (5) **108c** (5) **108c** (5)	36 18 2 5 2	93 >95 90 >95 >95

^a^Reaction conditions: catalyst, 55 °C. ^b^E = COOEt. ^c^Conversion determined by ^1^H NMR spectroscopy. ^d^80 °C.

**Figure 17:**
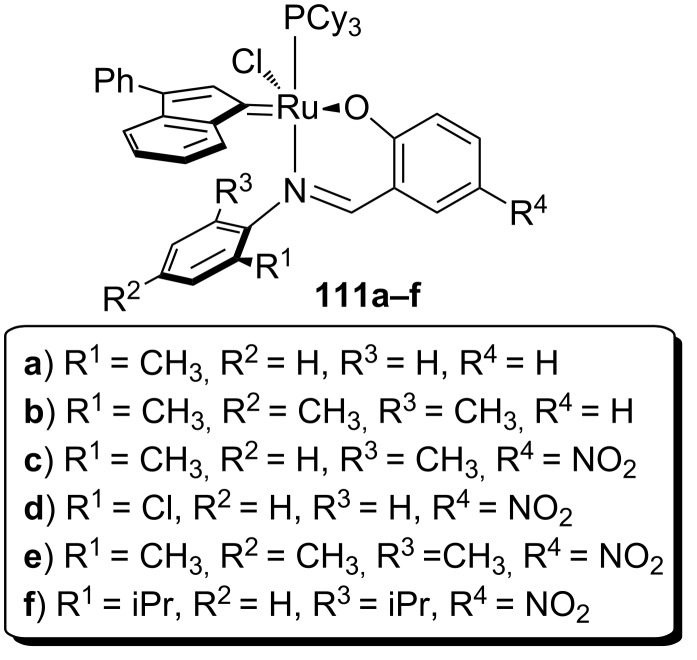
Veerport's indenylidene Schiff-base complexes.

In 2009, surely inspired by the aforementioned work, the Verpoort group reported a family of indenylidene Schiff base–ruthenium complexes (**111a–f**, Figure 17) for CM and RCM reactions in air [98]. They combined the higher thermal stability of indenylidene complexes and the tunability and stability of Schiff base ligands. These complexes were able to perform CM and RCM reactions in air with lower catayst loadings compared to **105a–f**, **106a–f**, **107a–f** and **111a–c**. RCM reactions proceeded smoothly using *N,N*-diallyltosylamide (**46**) giving, with all catalysts, quantitative yields. When a more challenging substrate (*N*-allyl-*N*-(methallyl)tosylamide, **79**) was used, a 24 h reaction time was needed in all cases, with the exception of **111d** (Table 8). This remarkable activity (higher than Hoveyda–Grubbs 2^nd^ generation catalyst, **33**) was due to the presence of the electron-withdrawing substituents on the Schiff base.

**Table 8:** RCM of *N*-allyl-*N*-(methallyl)tosylamide (**79**) with complexes **111a–f** in air.^a^

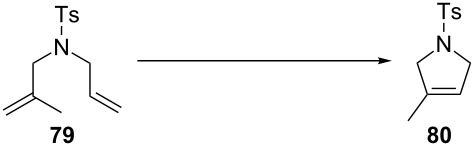			

Catalyst (0.5 mol %)	Yield over time		
	1 h	3 h	24 h

**111a**	18	37	51
**111b**	45	67	97
**111c**	14	37	87
**111d**	87	100	100
**111e**	36	68	97
**111f**	28	55	100

^a^Reaction conditions: catalyst, CH_3_Cl (0.1 M), 60 °C in air.

## Conclusion

Although metathesis-type reactions represent one of the most valuable strategies in modern organic synthesis, making this highly valuable tool more accessible and practical for routine use still remains a challenge. Ruthenium-based catalysts have been at the centre of recent advancements making possible their use in air, moreover these catalysts are becoming more and more stable, efficient and economically friendly with time. With the current development directed towards air and moisture stability and high performance, there is no doubt that more reports will push these reactivity/tolerance limits even further. As seen in this review, conducting metathesis-type reactions in air, in the presence of water and under high temperature has become more concrete, with several groups leading the charge [62,86].
